# A Case Report of Severe Dehydration Associated With Acute Kidney Injury Causing Acute ST-Segment Elevation Myocardial Infarction

**DOI:** 10.7759/cureus.25226

**Published:** 2022-05-22

**Authors:** Hosam Zaky, Sadeq Tabatabai, Parveez A Zarger, Jasem M Al Hashmi

**Affiliations:** 1 Cardiology Department, Dubai Hospital, Dubai Health Authority, Dubai, ARE

**Keywords:** acute kidney injury, case report, acute coronary syndrome, blood viscosity, myocardial infarction, dehydration

## Abstract

The initial electrocardiogram finding in the setting of acute myocardial infarction typically shows either persistent ST-segment elevation or non-ST-segment elevation. In young adults, when coronary angiography is performed, can further classify the patient with an occluded vessel and those with non-occluded coronary arteries. In these subgroups, myocardial infarction can be explained on the basis of coronary artery thrombosis, embolization, spontaneous coronary artery dissection, myocardial bridging, coronary aneurysms, ectasia, anomalous origin of coronary arteries coronary microvascular dysfunction, and vasospasm, or a combination of these factors.

We describe a 37-year-old male with a history of chest pain and electrocardiographic evidence of acute myocardial infarction who worked many hours under the sun before being presented to the emergency department. The initial laboratory tests showed evidence of acute kidney injury. He underwent a rescue coronary angiogram due to failed initial medical reperfusion therapy with Tenecteplase, which revealed occluded of the distal left anterior descending (LAD) artery with a minor lesion in proximal LAD and right coronary artery.

Our patient experienced acute myocardial infarction owing to severe dehydration. This case is important as it highlights that severe dehydration can be considered one of the triggering factors for acute myocardial infarction in young men who are at risk. Proper hydration could be a preventive measure.

## Introduction

In patients with acute myocardial infarction (AMI), the initial 12-lead electrocardiogram (ECG) typically shows either predominant ST-segment elevation or no predominant ST-segment elevation. In young adults, when a coronary angiogram is performed during myocardial infarction, can further be broadly categorized the patients into the group with an occluded vessel and those with angiographically normal coronary arteries [1].

While accelerated atherosclerosis and plaque rupture continues to be the most usual etiology of myocardial infarction, the pathophysiology of acute myocardial infarction in young patients remains uncertain. It can be explained on the basis of coronary artery thrombosis (especially in inherited or acquired hypercoagulability disorders), coronary embolization, spontaneous coronary artery dissection, myocardial bridging, coronary aneurysms, ectasia, anomalous origin of coronary arteries coronary microvascular dysfunction and vasospasm, or a combination of these factors [1,2].

As the likely mechanism of infarction, hypovolemia as a probable cause of coronary artery spasm leading to ST-segment elevation myocardial instruction (STEMI) has been reported [3]. Although coronary artery thrombosis can be seen in hypercoagulable states such as in the nephrotic syndrome, anti-phospholipid syndrome, cancer, and protein S and factor XII deficiencies [1,2], dehydration as a cause of coronary artery thrombosis triggering acute STEMI has been rarely previously described. Hereby, we report a case of acute STEMI that occurred in a severely dehydrated young adult male associated with acute kidney injury.

## Case presentation

A 37-year-old previously healthy male patient, with a history of prolonged exposure (many hours) to the sun before presenting to the emergency department (ED) with complaints of epigastric and lower chest pain associated with sweating, vomiting, generalized body aches, cramps, which were started approximately 90 minutes before arrival to ED. He has no significant past medical history and he was not on any regular medications.

His vital signs on arrival showed blood pressure (BP) 143/112 mmHg (BP location: right arm, patient position: lying), pulse 110 beats per minute (bpm), body temperature 37.1 °C (98.8 °F) (tympanic), respiratory rate 24 breaths per minute, blood oxygen saturation (SpO_2_) 96%, body mass index (BMI) 19.49 kg/m². Clinical examination was unremarkable apart from clinical evidence of dehydration with dry skin and lips. There were no clinical signs of heart failure.

**Figure 1:**
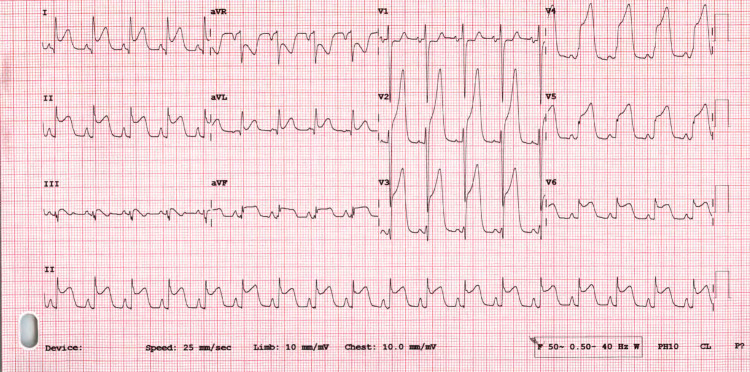
Electrocardiogram on arrival showing marked ST-segment elevation myocardial infarction mainly in anterolateral leads (leads V2-6, I, aVL, and II).

Initial resting ECG showed sinus rhythm with ST-segment elevation in anterolateral leads (Figure 1). He received a loading dose of dual antiplatelet therapy, and he was thrombolysed with Tenecteplase (TNK) in combination with enoxaparin according to the international protocol. As his initial laboratory tests revealed significant leukocytosis and polycythemia along with deranged renal function, thus he was resuscitated with intravenous normal saline as well.

**Figure 2:**
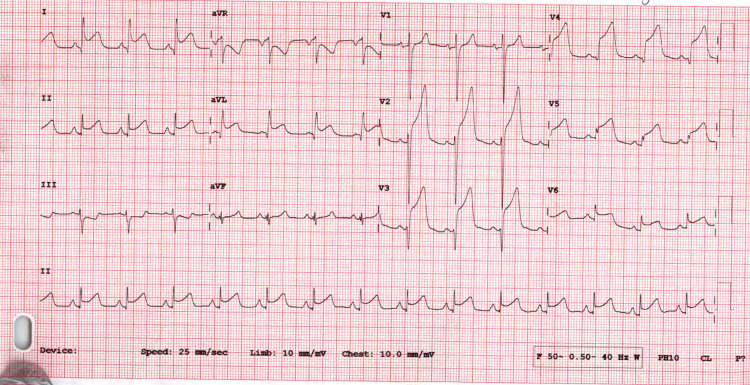
Electrocardiogram after thrombolysis showing evidence of failure of medical reperfusion therapy evident by persistent ST segment elevation in anterolateral leads.

**Figure 3:**
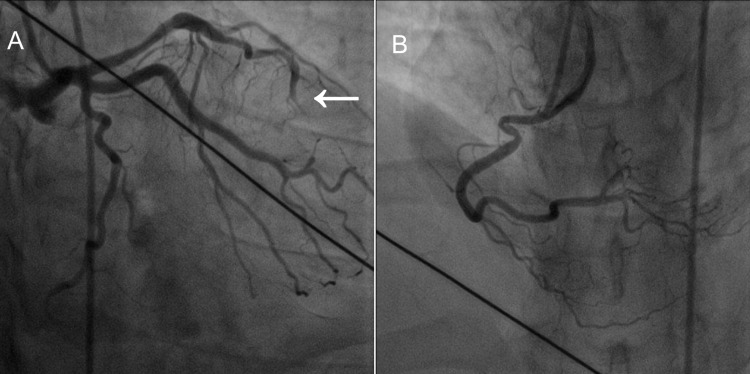
(A) Coronary angiogram showing non-significant proximal coronary lesion of left anterior descending artery with distal total occlusion (arrow). (B) Right coronary artery angiogram revealing non-significant lesion.

ECG at 90 minutes post TNK showed evidence of failed (unsuccessful) thrombolysis (Figure 2), for which he was taken for rescue coronary angiogram, which revealed occluded of distal left anterior descending (LAD) artery with a minor lesion in proximal LAD and right coronary artery (RCA) approximately 30%-40% (Figures 3A, 3B), hence coronary angioplasty was not performed. Coronary angiogram was completed using lowest necessary dose (about 40 mL) of nonionic, water-soluble radiographic contrast medium with coverage of continuous intravenous infusion of normal saline.

During hospital stay, a transthoracic echocardiogram showed normal left ventricular (LV) size, overall LV systolic function is moderately impaired with an ejection fraction (LVEF) of approximately 40%. There was apical LV hypokinesis. The diastolic filling pattern indicates impaired relaxation. Right ventricular systolic pressure is normal at < 35 mmHg.

Blood tests revealed a peak troponin T of 2,815 ng/L (reference range: <14 ng/L), peak creatine kinase MB isoenzyme (CK-MB) mass of 678.0 ng/mL, peak creatine phosphokinase (CPK) of 6,046 U/L, and N-terminal pro-brain natriuretic peptide (NT-proBNP) of 5,715 pg/mL. Total fasting cholesterol of 266 mg/dL, triglycerides of 312 mg/dL, high-density lipoprotein (HDL) of 57 mg/dL, low-density lipoprotein (LDL) of 147 mg/dL. Initial serum creatine of 3.0 mg/dL with estimated glomerular filtration rate (eGFR) of 25.4 mL/min/1.73m^2^, urea of 49.0 mg/dL, sodium of 132.0 mmol/L, potassium of 4.4 mmol/L, phosphate of 7.9 mg/dL, and calcium of 12.4 mg/dL. Initial white blood cell (WBC) count of 32.3*10^3^/µL, red blood cell (RBC) count of 8.06*10^6^/µL, a hemoglobin level of 21.4 g/dL, and hematocrit of 66.0%. Severe acute respiratory syndrome coronavirus 2 (SARS-CoV-2) reverse transcriptase polymerase chain reaction (PCR) assay of nasopharyngeal swab was negative.

The next day, as a result of acute kidney injury (serum creatinine peaked up to 6.0 mg/dL and urea to 88 mg/dL) and reduced urine output (around 200 mL/day), continuous veno-venous hemodialysis (CVVHD) was initiated for 18 hours, along with proper hydration, following which he made a good recovery. His kidney function normalized and he started passing a good amount of urine. Predischarge follow-up laboratory tests showed serum Creatinine of 0.8 mg/dL, Urea of 27 mg/dL, Sodium of 134 mmol/L, Phosphate of 3.3 mg/dL, and Calcium of 8.9 mg/dL. WBC count of 8.8*10^3^/µL, RBC count of 4.76*10^6^/µL, hemoglobin of 12.8 g/dL, and hematocrit of 39.3%. The patient was discharged home after full recovery of kidney function and normalization of his laboratory tests.

## Discussion

Chan et al. [4] have shown that the strongest and the most reliable association with fatal coronary heart disease was found with water intake (dehydration). Among men, univariate analysis showed a dose-response relation (p < 0.001) [4]. Compared with those drinking two or fewer glasses of water daily (low), subjects drinking from three to four glasses (medium) and five or more glasses (high) had relative risks of 0.65 and 0.46, respectively [4]. Whole blood viscosity (WBV), plasma viscosity, hematocrit, and fibrinogen are considered independent risk factors for coronary heart disease and can be elevated by dehydration [4].

WBV is the fundamental resistance of blood flow in the vessels. The markers of hemoconcentration and dehydration including hematocrit value, red blood cell counts, hemoglobin, and total plasma protein concentrations may influence WBV and can promote endothelial shear stress, endothelial inflammation, and vascular remodeling play important role in the initiation, acceleration, and progression of atherosclerosis [5,6].

A previous study showed in the setting of an acute coronary syndrome (ACS), hemorheological variables, especially higher hematocrit value (erythrocyte concentration) plays a role in the occurrence of STEMI and complete coronary artery occlusion, possibly by increasing the resistance of the microvasculature and decreasing the epicardial coronary blood flow [7].

AMI in males appears to be associated with increases in plasma viscosity regardless of the presence of ST-segment elevation. These findings indicate that reducing plasma viscosity may have clinical benefits for patients [8]. Hemorheological impairment, in particular hyper-viscosity, may unfavorably influence the long‐term prognosis of AMI in young adults [9]. In patients undergoing either urgent or elective PCI, hemorheological parameters might contribute to myocardial injury [10].

Unlike primary erythrocytosis or polycythemia vera in which there are elevated levels of three blood cells: red cells, white cells, and platelets, secondary polycythemia (also known as secondary erythrocytosis or erythrocythemia) due to cyanotic congenital heart disease, indicate increase red blood cells predominantly [11].

At extreme levels of hyper-viscosity due to secondary polycythemia, patients can be at risk for arterial thrombosis and myocardial infarction. The risk is probably lower than with primary erythrocytosis [12,13] but data are too scant for accurate quantification and limited to an insufficient number of published case reports [14-17] and, therefore, the relationship between hyper-viscosity and thrombosis is not clearly established in this group of patients.

The pathophysiology of thromboembolic events has not been fully explained in polycythemia vera, but many factors are involved including the increase in hematocrit and blood hyper-viscosity, increase platelet aggregation, thrombogenesis, and thrombocytosis, the presence of leukocytosis and intimal proliferation [12,14].

In secondary polycythemia due to cyanotic congenital heart disease, although the viscosity is increased, the other factors may be normal [18]. Indeed, Goldschmidt et al. [18] suggested that in some patients there may be reduced platelet adhesiveness and platelet survival is significantly shorter than in normal subjects. The results of this study also showed the peripheral destruction of the platelets is increased in patients with cyanotic congenital heart disease [18].

We propose that our patient suffered from acute myocardial infarction due to hemoconcentration and an increase in plasma viscosity associated with severe dehydration and acute kidney injury. This case is essential as it focuses that severe dehydration can be well-thought-out as one of the triggering factors for acute myocardial infarction in young men. The other possible trigger for AMI in our patients is accelerated atherosclerosis and soft plaque erosion and rupture.

We have given due consideration to the possibility of the acute renal impairment is due to type 1 cardiorenal syndrome, in which acute heart failure and a cardiogenic shock result in acute kidney injury [19], but we felt that this diagnosis is unlikely based on a careful history and physical examination. The significance of acute kidney injury in our patients is most likely due to severe dehydration rather than acute heart failure secondary to AMI. Proper hydration might be a preventive measure for acute myocardial infarction in laborers who are at risk. 

## Conclusions

We described a case of a young adult who presented with acute myocardial infarction and acute kidney injury secondary to severe dehydration due to prolonged exposure to the sun. This case is important as it highlights that severe dehydration can be considered a precipitating factor for an acute myocardial infarction in young men. Proper hydration could be a preventive measure for such events in laborers who are at risk.
